# Electronic acquisition of OSCE performance using tablets

**DOI:** 10.3205/zma000983

**Published:** 2015-10-15

**Authors:** Achim Hochlehnert, Jobst-Hendrik Schultz, Andreas Möltner, Sevgi Tımbıl, Konstantin Brass, Jana Jünger

**Affiliations:** 1 Universität Heidelberg, Kompetenzzentrum für Prüfungen in der Medizin/Baden-Württemberg, Heidelberg, Deutschland

**Keywords:** Quality assurance, OSCE, computer-based assessments, tablets, iPad, IT solution

## Abstract

**Background: **Objective Structured Clinical Examinations (OSCEs) often involve a considerable amount of resources in terms of materials and organization since the scores are often recorded on paper. Computer-assisted administration is an alternative with which the need for material resources can be reduced. In particular, the use of tablets seems sensible because these are easy to transport and flexible to use.

**Aim:** User acceptance concerning the use of tablets during OSCEs has not yet been extensively investigated. The aim of this study was to evaluate tablet-based OSCEs from the perspective of the user (examiner) and the student examinee.

**Method: **For two OSCEs in Internal Medicine at the University of Heidelberg, user acceptance was analyzed regarding tablet-based administration (satisfaction with functionality) and the subjective amount of effort as perceived by the examiners. Standardized questionnaires and semi-standardized interviews were conducted (complete survey of all participating examiners). In addition, for one OSCE, the subjective evaluation of this mode of assessment was gathered from a random sample of participating students in semi-standardized interviews.

**Results: **Overall, the examiners were very satisfied with using tablets during the assessment. The subjective amount of effort to use the tablet was found on average to be “hardly difficult”. The examiners identified the advantages of this mode of administration as being in particular the ease of use and low rate of error. During the interviews of the examinees, acceptance for the use of tablets during the assessment was also detected.

**Discussion: **Overall, it was found that the use of tablets during OSCEs was well accepted by both examiners and examinees. We expect that this mode of assessment also offers advantages regarding assessment documentation, use of resources, and rate of error in comparison with paper-based assessments; all of these aspects should be followed up on in further studies.

## Zusammenfassung

**Hintergrund: **OSCE-Prüfungen sind oft mit einem erheblichen Material- und Organisationsaufwand verbunden, da die Leistungserfassung üblicherweise auf Papier durchgeführt wird. Eine elektronisch unterstützte Durchführung stellt hierzu eine Alternative dar, mit der der Verbrauch materieller Ressourcen reduziert werden kann. Insbesondere erscheint hier der Einsatz von Tablets sinnvoll, da diese zudem leicht zu transportieren sind und damit flexibel eingesetzt werden können.

**Zielsetzung: **Die Nutzerakzeptanz der Verwendung von Tablets bei OSCE-Prüfungen wurde bislang allerdings nur wenig untersucht. Ziel dieser Studie war daher eine Evaluation Tablet-basierter OSCE-Prüfungen aus Sicht der Benutzer (Prüfer) und der geprüften Studierenden.

**Methodik: **Bei zwei OSCE-Prüfungen des Faches Innere Medizin der Universität Heidelberg wurde die Nutzerakzeptanz einer Tablet-basierten Durchführung (Zufriedenheit mit der Funktionalität) und die subjektive Anstrengung aus Sicht der Prüfer untersucht. Hierzu wurden standardisierte Fragebögen und halbstandardisierte Interviews eingesetzt (Vollerfassung aller teilnehmenden Prüfer). Zudem wurde bei einer der Prüfungen die subjektive Bewertung dieser Prüfungsvariante an einer Stichprobe teilnehmender Studierender mittels halbstandardisierter Interviews erhoben.

**Ergebnisse: **Die Prüfer waren mit der Tablet-Prüfungsvariante insgesamt sehr zufrieden. Die subjektive Anstrengung der Bedienung der Tablets wurde im Mittel als „kaum anstrengend“ empfunden. In den Interviews wurden insbesondere die einfache Handhabung und die geringe Fehleranfälligkeit von den Prüfern als Vorteile dieser Prüfungsvariante genannt. In der Befragung der geprüften Studierenden zeigte sich ebenfalls eine Akzeptanz der Tablet-Prüfungsvariante.

**Diskussion: **Insgesamt hat sich gezeigt, dass der Einsatz von Tablets in OSCE-Prüfungen sowohl von Prüfern als auch Studierenden gut angenommen wird. Es wird erwartet, dass diese Prüfungsvariante auch hinsichtlich Prüfungsdokumentation, Ressourceneinsatz und Fehleranfälligkeit gegenüber der papierbasierten Form Vorteile bietet, was Gegenstand weiterer Studien sein sollte.

## 1. Introduction

Objective Structured Clinical Examinations (OSCEs) are often administered during medical education to assess practical and communicative skills [8]. However, this type of testing requires a great amount of staff, organization and materials [9], [3]. Computer-assisted alternatives can be used to reduce the use of resources and are now generally recommended by the majority (Ottawa Conference 2010 [1]).

Computers can be specifically used during OSCEs by the examiner to enter scores and comment on evaluations, but using computers to conduct this type of assessment still often entails great effort and can pose various difficulties. Obstacles to using computers are primarily the inability to transport them easily and the risk that monitors or other components could block the assessor’s view of the testing situation. This is why the use of computers for OSCEs has not yet established itself. The use of tablets in the OSCE setting makes much more sense due to their suitable smallness of size (e.g. a half sheet of paper), portability, and flexibility in terms of use.

Overall, conducting an assessment using tablets offers the practical advantages of reducing paper consumption and the need for space when compared to paper-based and computer-based examinations, respectively. To implement this mode of assessment on a broader basis, it should not just be a more feasible method, but also well accepted by the examiner and examinee. The subjective acceptance of administering OSCEs using tablets has not yet been closely investigated. Two studies [14], [12], [13], in which the program eOSCE was used on iPads, point to a comparable or even higher level of acceptance in both student examinees and examiners for the tablet-based version in comparison with the paper-based one.

A module for administering OSCEs with tablets (tOSCE) was also developed at Heidelberg University in 2011 as part of the Item Management System (IMS) [4]. Its user interface hardly differs from the eOSCE used in the previous studies [14], [12], [13]. Thus it remained open how well tOSCE would be accepted since this could have been dependent on the assessment software used.

The aim of this study was to evaluate tablet-based assessment using the software tOSCE from the perspectives of the user (examiner) and the student examinee. For two OSCEs, the level of satisfaction regarding functionality and the subjective amount of effort was measured by means of a questionnaire (examiners only) and capturing general impressions in interviews (examiners and examinees). If the software in use here is also well received, then this would indicate a generalized acceptance of tablet-assisted OSCEs (assuming that adequate software is used).

## 2. Method

### 2.1 Issue and Study Design 

To evaluate the satisfaction with functionality, the subjective perception of ease and the general impressions of tablet-based OSCE administration, both quantitative data and general impressions were collected. This was done by means of two different questionnaires and the conduction of semi-standardized interviews. A total of two surveys were conducted: one for the 2012 summer semester (questionnaire for examiners) and another for the 2012-13 winter semester (questionnaires for examiners, interviews with students).

#### Procedure for the assessment and survey

2012 summer semester:

The evaluation of the tablet-based assessment took place during an OSCE in Internal Medicine at the Heidelberg University Hospital in the summer of 2012. In two OSCE circuits of 10 stations each, 184 students were assessed over the course of two days. The length of the individual assessments was five minutes; the time allotted for moving between stations was one minute.

Paper-based and tablet-based assessments were conducted in equal measure at the OSCE stations. In each circuit, the assessment at five of the stations was paper-based and at the other five tablet-based. At noon, the mode was switched at each station, so that examiners who used the paper-based method in the morning used the tablet at the same station in the afternoon, and vice versa. Each student was thus assessed on paper at five stations and at the other five with tablets (see table 1 (Tab. 1)).

After completing the assessments, the examiners were each given a questionnaire, addressing the level of satisfaction with the software and the perceived level of difficulty regarding tablet-based administration of the assessment. These are described in more detail below.

2012-13 winter semester:

Due to the positive results of the first examiner survey, the assessment was conducted exclusively with tablets during the following semester. As a technical improvement, a comment function with predefined text blocks was added to the assessment software. During this semester, the examiners were asked about the individual aspects of this new comment function, their experiences and suggestions for improvements (questionnaire). In addition, interviews were conducted with students (see table 2 (Tab. 2)).

#### 2.2 Recruiting the Examiners 

2012 summer semester:

A total of 37 instructors participated as examiners in the 2012 summer semester assessment; each received Survey 1. All participants underwent special training on how to use tablets (length of time was approximately 20-30 minutes). During the training session each examiner had the opportunity to do a complete test run of an assessment at the particular OSCE station using a checklist.

2012-13 winter semester:

A total of 35 examiners participated in the 2012-13 winter semester assessment; each received Survey 2. With the exception of four examiners (two female, two male), all of the examiners had participated in the OSCE during the previous summer. Once again, all examiners underwent tablet training, which also covered the new function for entering comments.

#### 2.3 Recruiting the Students

After completing each OSCE, a group of student examinees were surveyed about their impressions of the tablet-based OSCEs. Data was collected from a total of 19 randomly selected students (for practical reasons strict randomization was not possible).

#### 2.4 Description of the Questionnaires and Interviews

2012 summer semester:

General information was gathered from the examiners regarding demographics (age, sex), professional status (chief physician, senior physician, specialty physician or assistant physician), and prior knowledge concerning computer or iPad literacy as well as the conduction of OSCEs.

To evaluate the overall satisfaction with tablet-based administration of the OSCE, the following question was posed: How satisfied are you overall with digital administration of OSCEs? (1=dissatisfied, 2=mostly dissatisfied, 3=less satisfied, 4=satisfied, 5=very satisfied).

To measure the subjective level of difficulty associated with using the software, the internationally evaluated SMEQ questionnaire was used with a visual analog scale (VAS) ranging from 0 (not at all difficult) to 220 (exceptionally difficult) [15], (German version [2]).

With this questionnaire, information was collected about the subjectively perceived effort needed to find and select a candidate, enter and correct scores, view assessment results, use additional functions (timer), and enter comments.

In addition, the examiners responded to the open-ended questions: Do you have the impression that your conduct as an examiner changed when using a tablet, if so, in what ways? What do you consider are the advantages and disadvantages of using tablets to administer assessments?

2012-13 winter semester:

**Questionnaire for examiners:**

The questionnaire used for the winter semester also included the question regarding overall satisfaction (see above), along with the questions: In your estimation what is the need for training and the time needed for refresher training? Which comment functions did you use? What other improvements would you like to see? In your opinion, what are the major differences between table-based and paper-based assessment in terms of conducting an examination? For you, what are the major differences between tablet-based and paper-based assessment in terms of evaluating examinees?

**Interviewing the students:**

The student survey occurred immediately following the OSCE. A random selection of students were questioned in semi-standardized interviews about their impressions regarding any changes in the testing atmosphere and asked to give feedback comparing the two types of assessment administration. The following questions were posed: What are your perceptions of the tablet-based assessment? What consequences do you see for yourself? What was your overall impression of the assessment? Two raters qualitatively analyzed the responses in terms of content and classified them according to categories [10].

#### 2.5 Description of the Hardware (Tablet/WLAN)

Tablets were used to record the performance at the OSCE stations. This involved the use of portable, flat computers of a lightweight design with touchscreens and no keyboard [source: https://www.wikipedia.de/]. A laptop functioned as the server (see Figure 1 (Fig. 1)).

#### 2.6 Description of the software (tOSCE)

With the software application (tOSCE), the examiner uses a tablet as the medium for entering scores. tOSCE was developed as a quality assurance measure to limit the rate of error and the effort involved with analysis [5]]. In addition, the software offers the same advantages regarding system reliability and legal certainty as CAMPUS Prüfungsplayer, which has been in use since 2005 at a number of medical schools [11]. In order to support the largest selection of suitable tablets, the player was developed in a platform-neutral manner, meaning that the software can be used with the common tablet operating systems, such as iOS or Android. The following contains descriptions of the most important software features:

##### 2.6.1 Entry and export of the testing stations prior to the assessment

Just as with a paper-based OSCE, the test questions are entered into the Item Management System (IMS) and the examination is compiled [7]. The examination can then be printed out from the IMS not only in hardcopy form, but also exported to the tablets as a digital examination (see Figure 2 (Fig. 2)).

##### 2.6.2 Identification of the examinees using QR codes

Upon registering for the assessment, each student is assigned a QR code that can be printed from the server. The students bring these codes with them to the assessment. By pointing the tablet’s camera at the QR code, the examinee is immediately and accurately identified at each station, so that there is little risk of mistake and yet sufficient flexibility for dealing with last-minute switching of places among the students (in principle, there is also the possibility of identifying the station and the instructors through QR scanning).

##### 2.6.3 Entry of the score with a checklist or global rating

Responses can be evaluated with a checklist, whereby individual questions have been assigned one or more points in advance, or with global ratings, in which the examiners evaluate the overall impression of a particular aspect, for instance the communicative performance.

##### 2.6.4 Use of different comment features

Since the examiner is responsible for recording scores and documenting performance (primarily very poor or excellent performance), multiple means were developed for conveniently entering comments about exam performance. Comments can be entered using the integrated keyboard function, an externally connected keyboard, by means of handwritten notes (finger tipping or using a special tablet stylus), or by means of audio recording (integrated microphone).

To enter comments quickly and easily, standardized blocks of text were put together in coordination with the instructors. These texts can be entered very quickly during the assessment or added immediately afterwards during the time allowed for changing stations (see Figure 3 (Fig. 3)).

##### 2.6.5 Quality assurance after the assessment

After scoring each student performance, the examination is wrapped up by the examiner by double clicking on the appropriate button for finalizing the assessment. This not only saves the scores, but also creates a screenshot of the completed checklist. This increases legal validity since, along with the database entry, a photo of the information entered by the examiner is created allowing for uncomplicated and transparent review of the examination [4].

After the assessment, the scores are automatically transmitted by the tablets to the server via WLAN and these are immediately available for test statistical analysis.

## 3. Results

### 3.1. Survey of the Instructors

2012 summer semester:

Thirty-five of the 37 instructors (95%) filled out the questionnaire; 27 were male and 8 female (11 chief physicians, 11 senior physicians, 12 specialty physicians and one assistant physician). Most of the participants were computer literate (6 very good, 21 good, 8 average) and had experience with the OSCE (19 with more than 5 years, 15 with 1-4 years, one with none).

The instructors found the software to be very good and evaluated it very positively: the mean value for the response to the question about overall evaluation was 4.7 on a Likert scale of 1-5 (1=dissatisfied, 5=very satisfied) with a standard deviation of 0.5 (a one-time response of 3 was the lowest value).

When questioning the instructors about the subjective amount of effort (SMEQ), it was seen that this was very low for most of the questions (VAS from 0-220; 0=not at all difficult, 220=exceptionally difficult).

The average values for the functions to find and select candidate (21.1), enter and correct assessments (30.1), view assessment results (32.6), and use additional functions, such as the timer (34.6), all lie within the range “hardly to slightly difficult”. Only the function used to enter comments (64.2) was rated as “slightly to somewhat difficult” (see Figure 4 (Fig. 4)).

In response to the question asking if their testing behavior changed, the majority of examiners answered that they did not give the examinees lower scores when using tablets than when using paper. This is supported by the observation that the scores for the tablet-based stations did not significantly differ from those earned at the paper-based stations (unpublished data). The advantages listed by the examiners were the ease of handling, lower rate of error, better overview, and the time saved allowing for more time to observe the examinee. The disadvantage cited by examiners on the questionnaire for the 2012 summer semester was that entering comments was not as easy as it was for the paper-based assessment.

2012-13 winter semester:

For the second survey, 33 of the 35 instructors (94%) filled out the questionnaire, of which 27 were male and six female.

Again, a high level of satisfaction with the software was seen (20 were very satisfied, 12 satisfied, and one less satisfied). Using the same Likert scale used for the first survey (1=dissatisfied, 5=very satisfied), the second survey showed a mean value of 4.6 with a standard deviation of 0.5 (a “3” was seen once as the lowest value).

An average of 11.8 minutes and a standard deviation of 12.4 minutes were estimated as the time needed to demonstrate tablet operation (4x zero minutes as lowest value, 1x 60 minutes as highest).

In response to which comment function they had used (multiple responses possible) to comment on the assessment performance, 10 examiners said that they had used finger tipping, seven examiners answered that they had used the tablet keyboard, 14 examiners had used the predefined texts. Eleven examiners reported not having used the comment function at all.

When asked about the main differences between conducting assessments using tablets or paper, six instructors reported that the documentation was less subject to error, that they had more time for the examinee, and that it was possible to make better observations.

#### 3.2. Survey of the Students

The additional survey of a random sample of 19 students revealed that the students rated the tablet-based assessment very positively. The following aspects were mentioned specifically:

Better focus on the assessment: The instructors were able to follow the time exactly and thus manage the time for the assessment more fairly (1x). The examiners could focus more on the examinee (1x). During the assessment, the examiner was less distracted by the paperwork and totaling points and was better able to concentrate on the examinee (1x).

No computational errors: The scoring was more direct and fair, since the examiners don’t total the point values anymore, but rather the computer takes over the task of adding the points (1x). Examiners cannot enter any incorrectly added point values (2x).

Better transmission of information: Scanning in the examinee is convenient since there is more time for reading test questions (3x). The results are available much more quickly (1x).

## 4. Discussion

This study analyzed the level of satisfaction with functionality, the subjectively perceived amount of effort, and the general impressions and evaluations of tablet-based OSCE administration.

### 4.1 Survey of the Examiners

The survey of the examiners showed that tablet-based assessment was well received by the instructors. The level of satisfaction among instructors regarding the functionality of this assessment variant was very high. The subjective experience concerning difficulty and effort involved with various aspects of assessment administration were usually perceived to be low.

Finding and selecting a candidate was rated as the simplest operation, which can be explained by the use of QR codes to identify the students (this is a difference between the tOSCE software used here and eOSCE [12], [14], [13]).

Only the function for making comments was found to be more difficult to use (M 64 “slightly to somewhat difficult”). This could be due to the fact that, with a tablet, the comments had to be entered using the keyboard or by hand, possibly increasing the difficulty in comparison to paper-based assessment. The expansion of the comment function to include predefined texts led to an improvement in the following semester, as was seen in the feedback given by examiners.

A very large number of examiners indicated that they were better able to concentrate on the examinee. Indeed, the tablet-based variant does have several advantages when compared to an assessment done using a computer: since the tablet is small and can lie flat on a table, there is no monitor standing between the examiner and examinee, something which would compromise visibility. In addition, the examiner can change positions during an assessment and still be able to enter the points, an aspect primarily important for recording procedural skills. Due to a tablet’s portability, an examiner can observe the examinee from different angles and thus participate more actively in the assessment.

Overall, the survey of the examiners points out that routine use of tOSCE software is accepted by users and can be implemented without problems. This corresponds with the results of a Swiss study [12], [14], in which it was also shown that the use of tablets in OSCEs meets with a high level of acceptance on the part of examiners.

#### 4.2 Survey of the Students

The results of our student survey align with studies by Schmitz et al. [13], according to which electronic checklists as a method of documentation is positively rated by students and is accepted in a manner comparable to that of hardcopy checklists. In the interviews we conducted, students named other aspects that they valued, such as correct addition of the point values and the impression that the examiners were better able to concentrate on the student’s performance.

#### 4.3 Limitations of the Study

Since the assessment only covered one subject and was administered at only one medical school, broader analysis in the form of a systematic study would be desirable. In addition, no direct comparison of tablet-based and paper-based assessment was undertaken, but rather this was only indirectly addressed in some of the questions (e.g. the question about advantages and disadvantages of the tablet in relation to paper). In respect to the evaluation of the assessment performance, there was no difference found in the scores with tablets compared to earlier conventional assessment procedures using hardcopy checklists.

#### 4.4 Conclusion and Outlook

This study was able to show that tablet-based administration of OSCEs with the software tOSCE is accepted by examiners and examinees.

The use of tablets has the advantage of administering paper-free and flexible assessments. In terms of the future, further developments are conceivable for use in workplace-based assessments (e.g. encounter cards, mini-cex, DOPS) with even smaller devices, for instance those fitting in the pocket of a physician’s white coat.

Furthermore, to develop tOSCE it would certainly be helpful if it were used at other sites for other subject combinations. This would also indicate the extent to which the results seen here for tOSCE can be applied to OSCEs in general, regardless of the hospital or subject.

It is expected that tablet-based assessment also offers advantages in terms of completeness of documentation (automatic record of the assessment, examiner comments), use of resources, and rate of error in relation to the paper-based form. These aspects should be investigated in future studies.

## 5. Competing interests

The authors declare that they have no competing interests.

## Figures and Tables

**Table 1:**
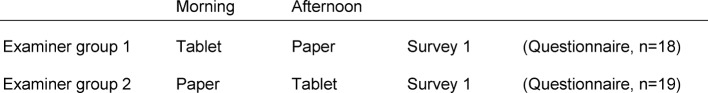


**Table 2:**
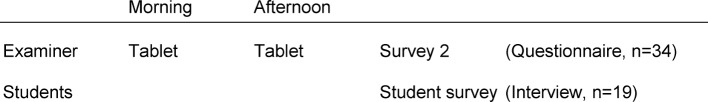


**Figure 1:**
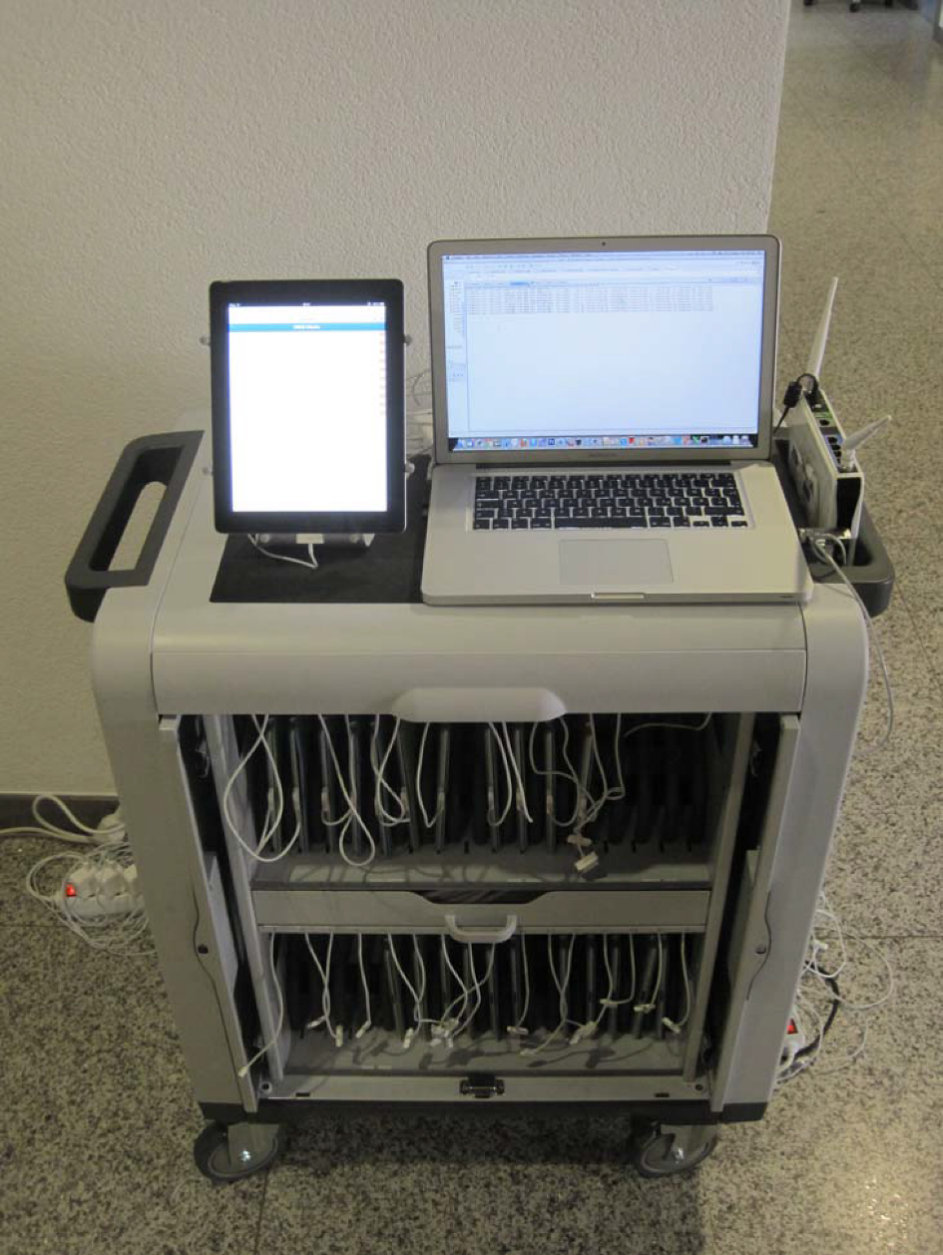
Tablet cart with 24 tablets and server (laptop), WLAN components: The tablets can be transported and charged simultaneously (the average battery life for a tablet depends on battery capacity, which can last up to eight hours). Prior to the assessment, the OSCE stations are saved to all the tablets at the same time; after the assessment, the scores are saved to the server.

**Figure 2:**
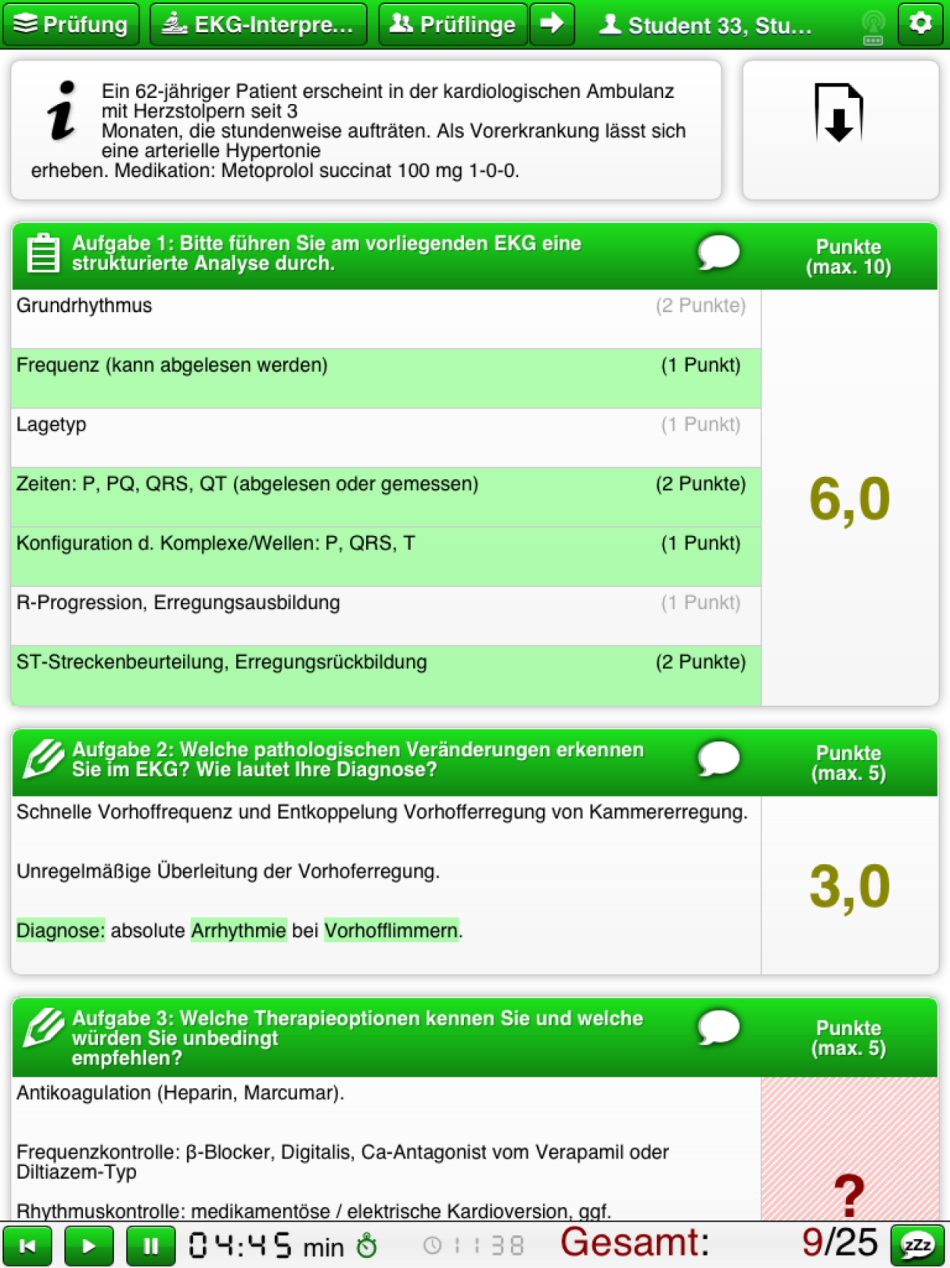
Tablet screenshot (checklist)

**Figure 3:**
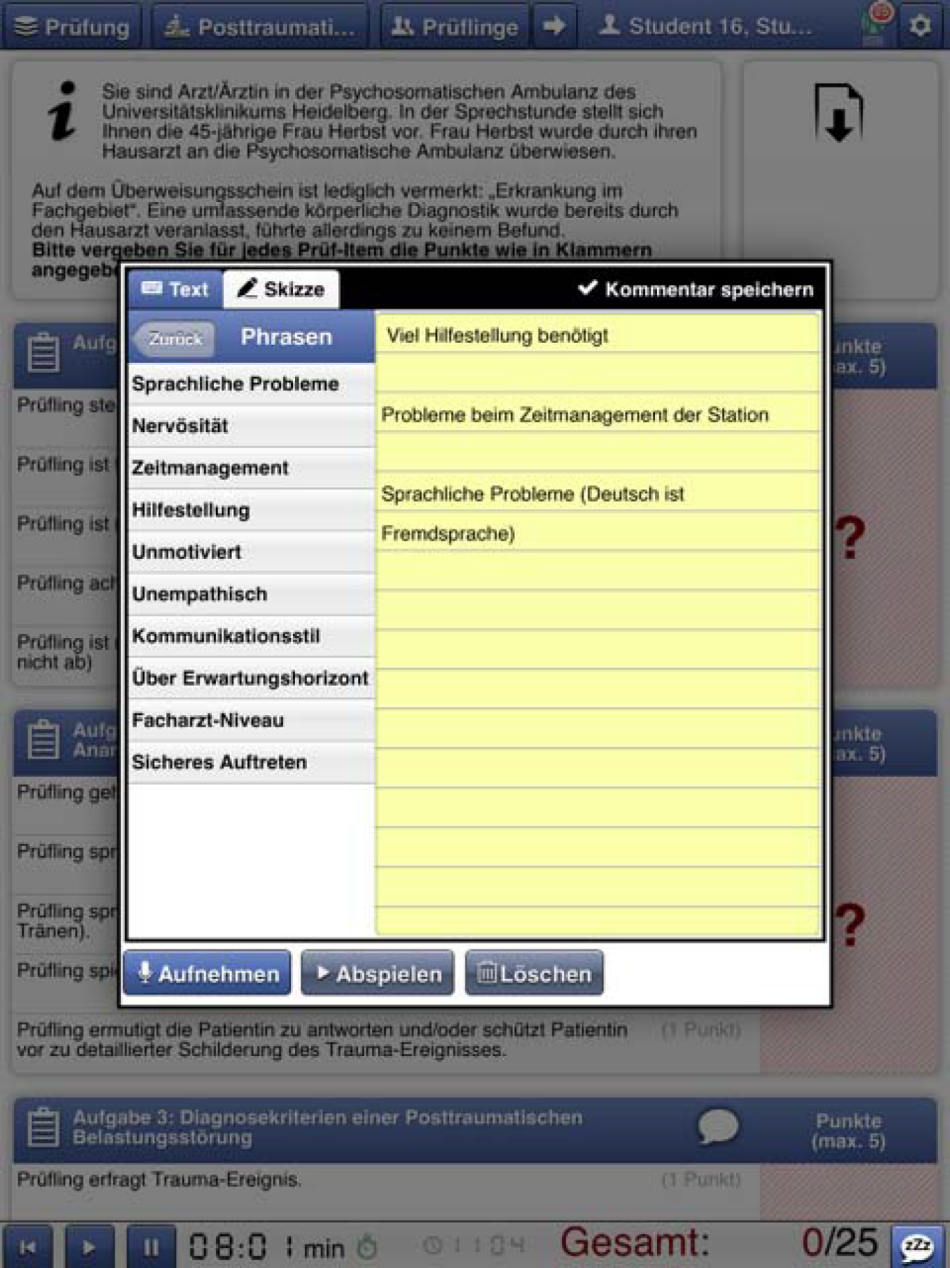
Tablet screenshot (comment function)

**Figure 4:**
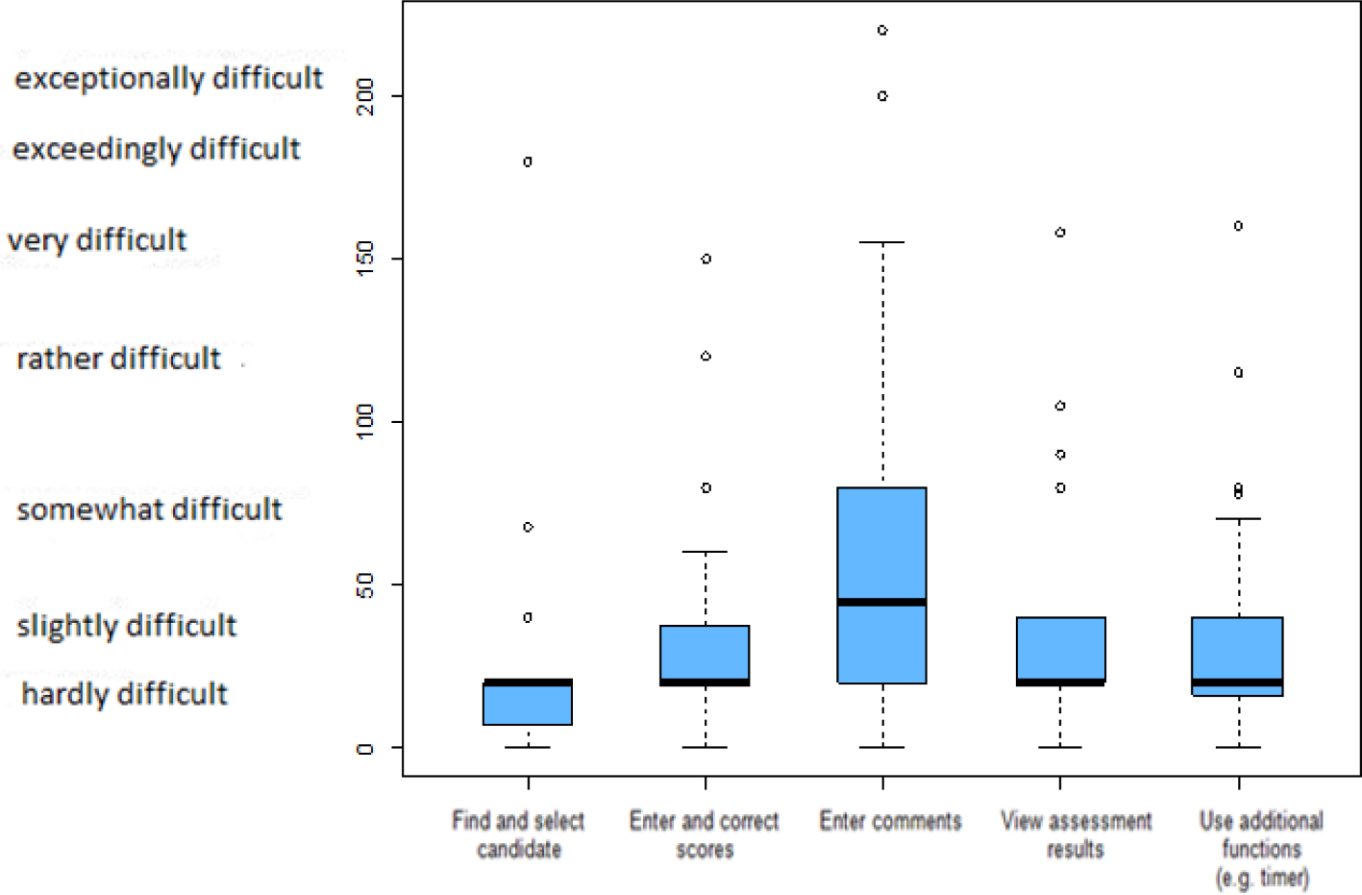
Estimation of the subjective effort as perceived by the instructors on the Subjective Mental Effort Questionnaire (SMEQ): minimum of 0 (not difficult), maximum of 220 (exceptionally difficult). Box plot depiction of mean and interval, n=35
